# Clinically Unapparent Infantile Thiamin Deficiency in Vientiane, Laos

**DOI:** 10.1371/journal.pntd.0000969

**Published:** 2011-02-22

**Authors:** Sengmanivong Khounnorath, Karen Chamberlain, Ann M. Taylor, Douangdao Soukaloun, Mayfong Mayxay, Sue J. Lee, Bounthom Phengdy, Khonsavanh Luangxay, Kongkham Sisouk, Bandit Soumphonphakdy, Khaysy Latsavong, Kongsin Akkhavong, Nicholas J. White, Paul N. Newton

**Affiliations:** 1 Department of Pediatrics, Mahosot Hospital, Vientiane, Lao People's Democratic Republic; 2 Wellcome Trust-Mahosot Hospital-Oxford Tropical Medicine Collaboration, Microbiology Laboratory, Mahosot Hospital, Vientiane, Lao People's Democratic Republic; 3 MRC Human Nutrition Research, Cambridge, United Kingdom; 4 Centre for Tropical Medicine, Churchill Hospital, University of Oxford, Oxford, United Kingdom; 5 Faculty of Postgraduate Studies, University of Health Sciences, Vientiane, Lao People's Democratic Republic; 6 Faculty of Tropical Medicine, Mahidol University, Bangkok, Thailand; The George Washington University Medical Center, United States of America

## Abstract

**Background:**

Beriberi occurs in Vientiane, Lao PDR, among breastfed infants. Clinical disease may be the tip of an iceberg with subclinical thiamin deficiency contributing to other illnesses. Thiamin treatment could improve outcome.

**Methodology/Principal Findings:**

A cohort of 778 sick infants admitted during one year without clinical evidence of beriberi were studied prospectively and erythrocyte transketolase assays (ETK) performed. Biochemical thiamin deficiency was defined both in terms of the activation coefficient (α>31%) and basal ETK activity <0.59 micromoles/min/gHb. Of the 778 infants, median (range) age was 5 (0–12) months, 79.2% were breastfed, 5.1% had α>31% and 13.4 % basal ETK<0.59 micromoles/min/gHb. Infants ≥2 months old had a higher frequency of biochemical markers of thiamin deficiency. Mortality was 5.5% but, among infants ≥2 months old, mortality was higher in those with basal ETK<0.59 micromoles/min/gHb (3/47, 6.4%) than in those with basal ETK≥0.59 micromoles/min/gHb (1/146, 0.7%) (P = 0.045, relative risk = 9.32 (95%CI 0.99 to 87.5)). Multivariate regression analysis indicated that infant age ≥2 months and fewer maternal years of schooling were independently associated with infant basal ETK<0.59 micromoles/min/gHb.

**Conclusions/Significance:**

Clinically unapparent thiamin deficiency is common among sick infants (≥2 months old) admitted to hospital in Vientiane. This may contribute to mortality and a low clinical threshold for providing thiamin to sick infants may be needed.

## Author Summary

Infantile beriberi, or clinical thiamin (vitamin B1) deficiency in infants, is a forgotten disease in Asia, where 100 years ago it was a major public health problem. Infants with this deficiency, commonly aged ∼ 2–3 months, present in cardiac failure but usually rapidly improve if given thiamin injections. It remains relatively common in Vientiane, Lao PDR (Laos), probably because of prolonged intra- and post-partum food avoidance behaviours. Clinical disease may be the tip of an iceberg with subclinical thiamin deficiency contributing to sickness in infants without overt clinical beriberi. We therefore recruited 778 sick infants admitted during one year at Mahosot Hospital, Vientiane, without clinical evidence of beriberi, and performed erythrocyte transketolase (ETK) assays. 13.4 % of infants had basal ETK<0.59 micromoles/min/gHb suggesting biochemical thiamin deficiency. Mortality was 5.5% but, among infants ≥2 months old, mortality was higher in those with basal ETK<0.59 micromoles/min/gHb (3/47, 6.4%) than in those with basal ETK≥0.59 micromoles/min/gHb (1/146, 0.7%) (P = 0.045, relative risk = 9.32 (95%CI 0.99 to 87.5)). We conclude that clinically unapparent thiamin deficiency is common among sick infants (≥2 months old) admitted to hospital in Vientiane. This may contribute to mortality and a low clinical threshold for providing thiamin to sick infants may be needed.

## Introduction

Beriberi, or clinically apparent thiamin deficiency, may present with a variety of syndromes including peripheral neuropathy, myocardial dysfunction, encephalopathy, hypoglycemia and lactic acidosis. With the advent of mechanical rice milling in the late 19^th^ century, beriberi became a dominant public health problem in Asia, responsible for a considerable mortality, especially amongst infants [1]–[8]. This stimulated a large research effort and identification of the aetiology led to changes in diet, supplementation and targeted health programs to ensure adequate thiamin intake. The incidence of thiamin deficiency in the more accessible, wealthier parts of Asia declined and interest in the disease waned. However, there are recent reports suggesting that it remains an important public health problem in Asia, especially amongst more vulnerable people, such as refugees [8]–[10], the elderly [11] and infants [10], [12], [13]. Treatment with parenteral thiamin is simple, inexpensive and highly effective but supplementation has been much more contentious [14]). In the Lao PDR (Laos), beriberi was noted in 1930 [15] and acknowledged as an important disease in the 1960s and 1970s [16], [17]. It was rediscovered in the early 1990s as an important cause of infant death in the capital, Vientiane [13], [18].

Thiamin deficiency is conventionally assessed using functional assays for the thiamin-dependent erythrocyte transketolase enzyme in washed red cells. The activation coefficient (α) is the ratio of *in vitro* erythrocyte transketolase activity (ETK) after thiamin pyrophosphate has been added minus the basal ETK before thiamin pyrophosphate has been added, to the basal ETK, expressed as a percentage. Higher α coefficients represent greater degree of thiamin deficiency. However, the α coefficient may not be the appropriate measure of thiamin deficiency in young infants [22] as, with very low exposure to thiamin *in utero* and post-partum, the capacity of ETK to have its activity augmented may be reduced, underestimating the frequency of biochemical thiamin deficiency [5], [19]–[21]. Indeed, at Mahosot Hospital, Vientiane, Laos basal ETK is a better discriminator between infants with and without clinical beriberi than α and may be the most relevant measure in these chronic deficiency states [22].

Overt infantile beriberi is relatively easy to diagnose but may be the tip of a much larger iceberg of deficiency. A significant proportion of infants admitted with other diseases, such as acute respiratory infection or diarrhoea, may also have clinically unapparent thiamin deficiency contributing to the illness, and thiamin treatment may improve their outcome. An autopsy study 100 years ago demonstrated that many infants with post-mortem evidence for beriberi were misdiagnosed with other conditions [23]. That clinically important but clinically unapparent thiamin deficiency may occur has been suggested in China [19], [24], [25], the Philippines [23], Australia [26], United Kingdom [27], Thailand [28], in Africa [19], [29], Jamaican children with malnutrition [30], the critically ill [31], in psychiatric patients [32] and in the disadvantaged [33] and elderly [34]. In rural southern Laos 30% of older children and adults presenting with malaria had evidence for biochemical thiamin deficiency [35]. Infectious diseases, such as typhoid and malaria, may precipitate beriberi [6], [25], [35], [36]. An increase in body temperature by 1 °C increases basal metabolic rate by 10%, increasing the utilization of thiamin [5]. Therefore, in societies with infantile beriberi, infectious diseases in infants and their mothers may contribute to clinically unapparent but important thiamin deficiency or frank beriberi. We therefore measured the ETK of infants admitted at a hospital in Vientiane to determine what proportion of sick infants without clinical beriberi had biochemical thiamin deficiency.

## Methods

### Ethics

Ethical approval was granted by the Ministry of Health, Laos and the Oxford Tropical Research Ethics Committee. Infants and their mothers were included if the mothers gave witnessed informed oral consent. This was thought to be the most appropriate mode for the Lao situation in 2002 by the two IRBs as the study was a simple observational design with minimal risk. Mothers were given an information sheet describing the study and informed oral consent was documented by the signature of someone who was not a member of the study team.

### Study site, patients and clinical procedures

We included all infants who had no overt clinical evidence of beriberi and were admitted to the paediatric service (57 beds) at Mahosot Hospital, a 400 bed primary-tertiary hospital in Vientiane, the capital of Laos. All infants were examined and treated according to hospital guidelines. We did not formally define beriberi but asked physicians to ensure that they only recruited infants whom they believed did not have beriberi. In this hospital paediatricians are very aware of thiamin deficiency and readily treat with thiamin. A 1.8 ml venous blood sample in lithium heparin was taken from the infant. If beriberi was suspected after recruitment, parenteral thiamin (50 mg) was given. Infants were recruited only on the first admission if they were admitted more than once during the study period.

### Laboratory procedures

Immediately after collection the lithium heparin anticoagulated blood was centrifuged and washed in phosphate buffered saline three times, with removal of the buffy coat initially and after each wash. Washed red cell samples were stored at −30 °C for a maximum of 3 months and then at −70 °C until shipment to the UK on dry ice. ETK assays were performed at two laboratories. In Oxford (patients 1–394) the assay was performed by a modification of the nicotinamide-adenine dinucleotide dependent method with ribose-5-phosphate as the substrate [36], [37] except that samples were collected into acid citrate dextrose by Krishna *et al.* [36]. Because of retirement of the machine in the first laboratory, the assay was switched to Cambridge, where an adaptation of the method of Vuilleumier *et al.* [38] was used (patients 395–778). A Cobas Fara (Roche Co., Gipf-Oberfrick, Switzerland) was used in both laboratories and at both sites α was calculated as: ((ETK Activated - ETK basal) /ETK basal) x100. Haemoglobin concentrations were not assayed in Oxford and therefore basal ETK could not be expressed in micromoles/min/gHb for these 394 sample triplets. The method in Oxford measured ETK activity in the presence and absence of TPP [37] whilst that in Cambridge [38] pre-incubated the sample and TPP before the assay reagents are added. The coefficients of variation for the ETK assays in Oxford and Cambridge were 9.0–10.0 and 5.1–7.7, respectively. Different upper reference ranges for α are described and we have used the more conservative α of >31% [36] as defining definite severe biochemical deficiency. Storage of *ex vivo* human samples for 18 months at −70 °C does not appear to affect α values [35], [36]. A case control study, also at Mahosot Hospital [22], suggested that the best discriminator between infants with and without clinical beriberi was a basal ETK<0.59 micromoles/min/gHb. We therefore also compared infants with and without basal ETK<0.59 micromoles/min/gHb.

### Statistical analysis

Data were analyzed using Stata (v10, StataCorp.). Comparisons between 2 groups (α>31 versus α≤ 31 and basal ETK<0.59 micromoles/min/gHb versus basal ETK≥0.59 micromoles/min/gHb) were made by the Mann-Whitney *U*, Student's *t*, Pearson's chi-squared, and Fisher's exact tests, as appropriate. Because of the multiple comparisons, a Bonferroni adjusted p-value conservatively rounded down to <0.02 was used. Significant factors from the univariate analysis were then included in two separate multivariate models to identify independent predictors of infantile beriberi using the α coefficient and basal ETK. Using a stepwise selection procedure, only variables that were significant at P<0.05 were retained in the final model. Independent predictors for mortality were also identified. We categorized infants as aged <2 and ≥2 months as a secondary infant mortality peak at 3–4 months of age is associated with a high incidence of infantile beriberi in a population [39], [40]. We have attempted to report the study according to the STROBE guidelines [41].

## Results

### Patients

Between January 7th 2003 and 2004 1,030 infants were admitted to the three pediatric wards. Their median (range) age was 4 (0–12) months and 595/1,023 (58.2%) were male. Seven hundred and seventy-eight (75.5%) infants were recruited; reasons for exclusion of 252 infants were: thiamin given before recruitment (34.1%), infant discharged (19.8%) or died (19.0%) before assessment, insufficient blood sample volume (15.9%), declined consent (6.3%), readmission (3.6%), responsible physician disagreed with recruitment (0.8%) and inability to take a blood sample (0.4%).

The median (range) age of the 778 infants recruited was 5 (0–12) months (Table 1). Patients were recruited on the general pediatric ward (26.6%), pediatric infectious disease ward (30.6%) and on pediatric intensive care (42.8%). Of 771 mothers with addresses recorded, 79.8% and 16.1% had homes in Vientiane City and Vientiane Province, respectively. The majority of infants (79.2%) were breastfed but 32.2% were both breast and bottlefed. Fever (≥37.5 °C), dyspnoea, central cyanosis and cold fingers were present in 52.8, 39.3, 10.4 and 17.9 % of infants, respectively. The median (range) daily maternal cash expenditure was 19,000 (0–60,000) Lao kip, equivalent to median (range) 2.4 (0–7.5) $US in December 2010. Mothers ate predominantly glutinous (sticky) rice, with a median (range) of 3 (0–4) meals/day, rather than non-glutinous rice.

**Table 1:** Demographic and clinical features of 778 infants without clinical beriberi.

Variable	All	Alpha≥25%	Alpha>31%	P c	Basal ETK≥0.59 micromoles/min/gHB	Basal ETK<0.59 micromoles/min/gHB	P d
**Infant Demography**	778	101 (13.0)	40 (5.1)		331 (86.6)	51 (13.4)	
Infant age/months a	5.0 (0–12)	6.0 (0–12)	6.0 (0–12)	0.07	1.0 (0–12)	7.1 (0–12)	<0.0001
Infant age ≥2 months	505 (64.9)	83 (82.2)	35 (87.5)	0.002	147 (44.4)	47 (92.2)	<0.001
Male	446/771 (57.9)	58/99 (59.0)	24/39 (61.5)	0.63	184/327 (56.3)	33/50 (66.0)	0.20
Died or taken home severely ill	43/777 (5.5)	4/101 (4.0)	1/40 (2.5)	0.39	24/330 (7.3)	5 (9.8)	0.53
Infants ≥2 months died or taken home severely ill	8/504 (1.6)	2/83 (2.4)	0/35	0.44	1/146 (0.7)	3/47 (6.4)	0.045
Thiamin given after recruitment	150/765 (19.6)	22/100 (22.0)	12/40 (30.0)	0.09	47/323 (14.6)	14 (27.5)	0.02
Duration in hospital/days a	3 (0–61) ^777^	3 (0–61)	4 (0–61)	0.04	3 (0–32) ^330^	3 (0–61)	0.52
**Infant Discharge diagnosis**							
Discharge diagnosis Diarrhoea	238 (30.6)	35 (34.7)	12 (30.0)	0.93	52 (15.7)	12 (23.5)	0.16
Discharge diagnosis Pneumonia	191/777 (24.6)	27 (26.7)	16 (40.0)	0.02	75/330 (22.7)	21 (41.2)	0.005
Discharge diagnosis Sepsis	94 (12.1)	12 (11.9)	3 (7.5)	0.36	51/330 (15.5)	5 (9.8)	0.29
Discharge diagnosis Prematurity	82/777 (10.6)	3 (3.0)	1 (2.5)	0.09	57/330 (17.3)	0	0.001
**Infant Clinical Features**							
Breast fed	613/774 (79.2)	96/101 (95.1)	38/40 (95.0)	0.01	229/328 (69.8)	49 (96.1)	<0.001
Both breast and bottle fed	249/774 (32.2)	27/101 (26.7)	11/40 (27.5)	0.52	82/328 (25.0)	10 (19.6)	0.40
Given rice water	327/774 (42.3)	40/101 (39.6)	15/40 (37.5)	0.53	76/328 (23.2)	11 (21.6)	0.80
Given chewed rice	239/774 (30.9)	33/101 (32.7)	11/40 (27.5)	0.64	71/328 (21.7)	15 (29.4)	0.22
Lethargic	224/774 (28.9)	32/100 (32.0)	8/39 (20.5)	0.23	73/328 (22.3)	7/50 (14.0)	0.18
Not feeding	262/773 (33.9)	35/100 (35.0)	16/39 (41.0)	0.33	75/327 (22.9)	15/50 (30.0)	0.28
Vomiting	333/774 (43.0)	59/100 (59.0)	25/39 (64.1)	0.006	98/328 (29.9)	28/50 (56.0)	<0.001
Dyspnoea	304/774 (39.3)	52/100 (52.0)	23/39 (59.0)	0.01	136/328 (41.5)	31/50 (62.0)	0.0006
Pulse/min b	122.1 (120.5–123.6) ^750^	116.2 (111.8–120.5) ^98^	116.8 (109.9–123.8) ^37^	0.14	128.1 ^312^ (125.7–130.6)	117.7 ^46^ (111.5–123.8)	0.003
Temperature °C b	37.5 (37.5–37.6) ^769^	37.7 (37.5–38.0) ^100^	37.7 (37.3–38.1) ^39^	0.36	37.4 ^325^ (37.2–37.5)	37.6 ^49^ (37.2–37.9)	0.27
Temperature >37.5 °C	406/769 (52.8)	59/100 (59.0)	20/39 (51.3)	0.85	155/325 (47.7)	24/49 (49.0)	0.87
Respiratory rate/min b	50.2 (50.0–51.4) ^541^	49.9 (46.0–53.8) ^64^	52.7 (45.2–60.2) ^24^	0.37	51.0 ^262^ (49.4–52.6)	48.3 ^33^ (42.8–53.7)	0.28
Peripheral oedema	25/772 (3.2)	4/100 (4.0)	1/39 (2.6)	0.81	2/327 (0.6)	0/50	0.58
Central cyanosis	80/772 (10.4)	16/100 (16.0)	7/39 (18.0)	0.11	40/327 (12.2)	6/50 (12.0)	0.96
Peripheral cyanosis	151/772 (19.6)	28/100 (28.0)	12/39 (30.8)	0.07	54/327 (16.5)	8/50 (16.0)	0.93
Fingers cold	138/772 (17.9)	22/100 (22.0)	17/39 (43.6)	<0.001	36/327 (11.0)	9/50 (18.0)	0.16
Respiratory distress	76/772 (9.8)	13/100 (13.0)	6/39 (15.4)	0.23	34/327 (10.4)	5/50 (10.0)	0.93
**Infant - Biochemistry**							
Basal erythrocyte transketolase activity micromoles/min/gHB^a^	0.84 (0.2–3.24) ^382^	0.50 (0.25–1.25) ^38^	0.37 (0.25–0.88) ^22^	<0.0001	---------------	-------------	--------
Basal transketolase activity <0.59 micromoles/min/gHB	51/382 (13.4)	28/38 (73.7)	19/22 (86.4)	<0.001	---------------	--------------	--------
Activated erythrocyte transketolase activity micromoles/min/gHB a	0.95 (0.23–3.42)^382^	0.71 (0.44–1.85) ^38^	0.62 (0.46–1.85) ^22^	<0.0001	-------------	------------	-------
Activated erythrocyte transketolase activity <0.74 micromoles/min/gHB	58/382 (15.2%)	21/38 (55.3)	16/22 (72.7)	<0.001	-------------	------------	-------
Alpha % a	13.7 (−13.2 to 110.1) ^778^	-------------------	-------------------	-----	10.7 (−2.6 to 110.1)	27.3 (−9.6 to 81)	<0.0001
Alpha≥25 %	101/778 (13.0)	-------------------	-------------------	-------	10 (3.0)	28 (54.9)	<0.001
Alpha>31 %	40/778 (5.1)	-------------------	-------------------	-------	3 (0.9)	19 (37.3)	<0.001
**Maternal Demography**							
Age/years b	26.7 (26.2–27.2) ^733^	26.6 (24.6–28.5) ^94^	25.7 (23.7–27.8) ^38^	0.36	26.9 ^309^ (26.2–27.6)	26.3 ^48^ (24.5–28.3)	0.58
% rice farmers	55/764 (7.2)	13/98 (13.3)	8/38 (21.1)	0.001	31/322 (9.6)	9/47 (19.2)	0.05
Lao Loum ethnicity e	662/773 (85.6)	77/99 (77.8)	28/38 (73.7)	0.03	282/328 (86.0)	30/49 (61.2)	<0.001
Lao Sung ethnicity f	98/773 (12.7)	21/99 (21.2)	10/38 (26.3)	0.01	38/328 (11.6)	18/49 (36.7)	<0.001
Gravidity a	2 (1–13) ^761^	2 (1–10) ^98^	2 (1–10) ^38^	0.50	2 (1–13) ^319^	2 (1–10) ^47^	0.04
Mother body weight/kg a	50 (35–87) ^746^	49 (38–75) ^96^	47 (40–69) ^37^	0.02	50 (38–76) ^308^	50 (41–78) ^45^	0.30
Daily expenditure/kip a	19,000 (0–60,000) ^759^	20,000 (1,400–60,000) ^97^	18,500 (8,000–56,000) ^38^	0.3	20,000 ^323^ (6,000–50,000)	18,000 ^47^ (0–40,000)	0.03
Years of schooling a	8 (0–16) ^748^	5 (0–11) ^95^	5 (0–11) ^38^	0.002	8 (0–16) ^318^	4 (0–11) ^46^	0.0005
Enough cash for daily expenditure	3/772 (0.4)	1/99 (1.0)	0/38	0.69	2/329 (0.6)	0/47	0.59
**Maternal clinical features & diet**							
Weak	130/768 (16.9)	16/98 (16.3)	6/38 (15.8)	0.85	47/326 (14.4)	5/49 (10.2)	0.43
Anorexia	152/767 (19.8)	16/98 (16.3)	7/38 (18.4)	0.83	34/325 (10.5)	2/49 (4.1)	0.16
Number meals sticky rice/day a	3 (0–4) ^768^	3 (0–4) ^97^	3 (0–4) ^38^	0.53	3 (0–4) ^326^	3 (0–4) ^49^	0.88
Number meals boiled rice/day a	0 (0–7) ^767^	0 (0–3) ^97^	0 (0–3) ^38^	0.71	0 (0–7) ^326^	0 (0–3) ^49^	0.89
Number hours soaked rice a	6 (0–15) ^768^	6 (0–12) ^97^	5 (0–12) ^38^	0.53	5 (0–11) ^326^	5 (0–8) ^49^	0.48
Number meals fish paste/day a	0 (0–7) ^768^	0 (0–3) ^97^	0 (0–3) ^38^	0.95	0 (0–7) ^326^	0 (0–1) ^49^	0.23
Number betel nuts/day a	0 (0–1) ^768^	0 ^97^	0/38	0.82	0/326	0/49	----
Consumed alcohol since gave birth	41/770 (5.3)	5/98 (5.0)	1/38 (2.6)	0.45	6/326 (1.8)	0/49	0.34
Food avoidance since gave birth	321/776 (41.4)	44/100 (44.0)	21/39 (53.9)	0.10	153/330 (46.4)	26/50 (52.0)	0.46
Eaten pork since gave birth	572/770 (74.3)	84/98 (85.7)	32/38 (84.2)	0.15	209/326 (64.1)	45/49 (91.8)	<0.001
Eaten chicken since gave birth	646/770 (83.9)	93/98 (94.9)	36/38 (94.7)	0.06	238/326 (73.0)	47/49 (95.9)	<0.001
Eaten fish since gave birth	582/770 (75.6)	86/98 (87.8)	33/38 (86.8)	0.10	209/326 (64.1)	44/49 (89.0)	<0.001
Eaten fruits since gave birth	656/770 (85.2)	89/98 (90.8)	33/38 (86.8)	0.94	251/326 (77.0)	45/49 (91.8)	0.02
Eaten vegetables since gave birth	626/770 (81.3)	88/98 (89.8)	35/38 (92.1)	0.08	232/326 (71.2)	46/49 (93.9)	0.001
Consumed milk products since gave birth	571/770 (74.2)	69/98 (70.4)	23/38 (60.5)	0.05	208/326 (63.8)	27/49 (55.1)	0.24

a  =  median (range);

b  =  mean (95%CI);

c  =  comparison of Alpha>31% with Alpha≤31%;

d  =  comparison of Basal ETK≥0.59 with <0.59 micromoles/min/gHb;

e  =  ‘Lao Loum’ or ‘lowland Lao’ are now classified as within the ‘Tai-Kadai ethno-linguistic family’ see [55];

f  =  ‘Lao Sung’ or ‘highland Lao’ are now classified as within the Hmong-Mien Group’ see [55].

The predominant discharge diagnoses were diarrhoea (30.6%), pneumonia (24.6%), sepsis (12.1%) and prematurity (10.6%). Parenteral thiamin was given to 19.6% of infants after recruitment but before discharge. The overall mortality (died in hospital or taken home critically ill, not expected to survive) was 92/1,029 (8.9%). For infants recruited to the study mortality was 43/777 (5.5%).

### Erythrocyte transketolase activity and activation coefficients

**Figure 1:**
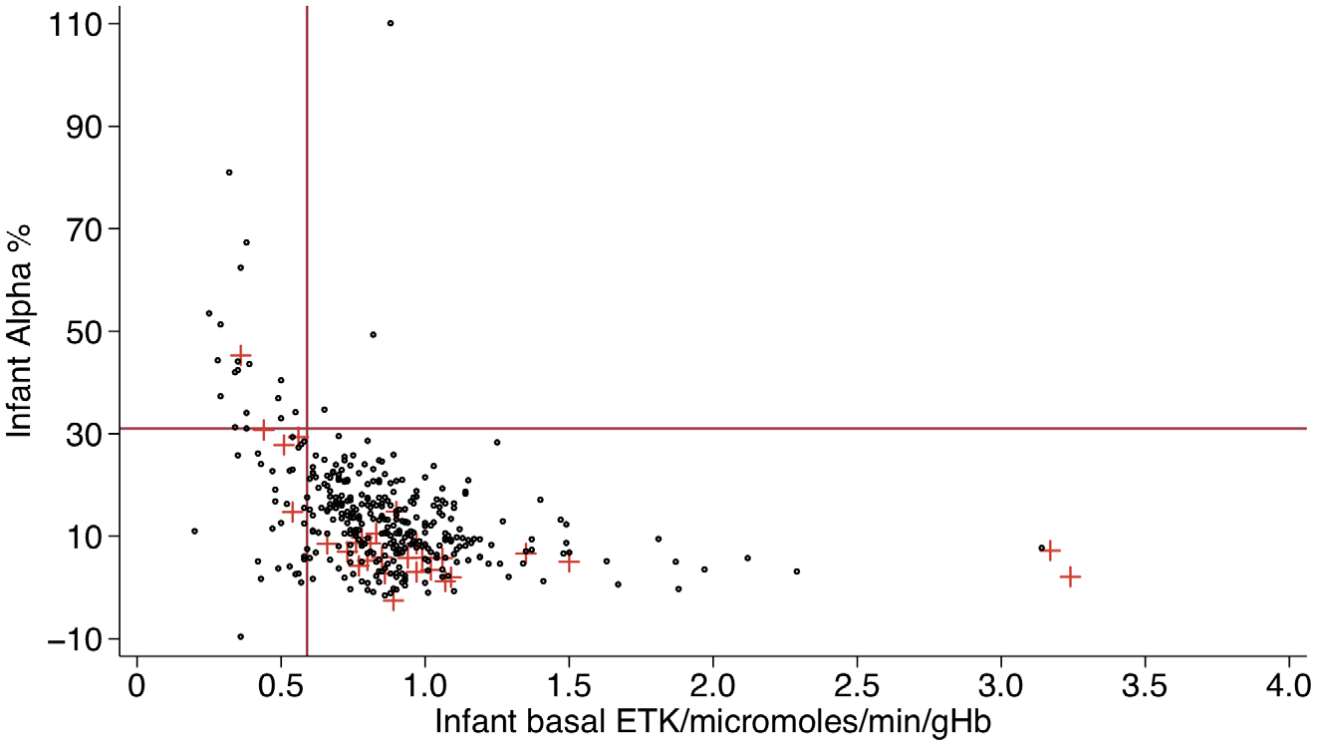
Relationship between α and basal ETK for 382 infants without clinical evidence of beriberi. Red crosses represent infants who died, The vertical and horizontal red lines represent the lower bound for a normal basal ETK (≥0.59 micromoles/min/gHB) and upper bound for a normal α (< = 0.31 %).

The median (range) α for 778 infants was 13.7 (−13.2 to 110.1) % (Table 1). The median (range) basal ETK and activated ETK for the 382 infants for whom these was measured were 0.84 (0.20–3.24) and 0.95 (0.23–3.42) micromoles/min/gHb, respectively. The α coefficient and basal ETK were inversely correlated (Spearman's rho = −0.46, p = <0.001, Fig. 1). Forty (5.1%) infants had an α>31% and 51/382 (13.4%) of infants had a basal ETK<0.59 micromoles/min/gHb (Table 1); 19/51 (37.3%) infants had basal ETK<0.59 micromoles/min/gHb and α>31% but 3/22 (13.6%) infants had α>31% and a basal ETK≥0.59 micromoles/min/gHb.

Of the 40 infants with α>31%, 30% received thiamin before discharge, in comparison to 19.3% of those with α≤31% (P = 0.09). Of the 51 infants with a basal ETK<0.59 micromoles/min/gHb, 27.5 % received thiamin before discharge, in contrast to 14.6% of those with a basal ETK≥0.59 micromoles/min/gHb (P = 0.02), suggesting that subtle clinical evidence of thiamin deficiency may have been recognized after admission.

**Figure 2:**
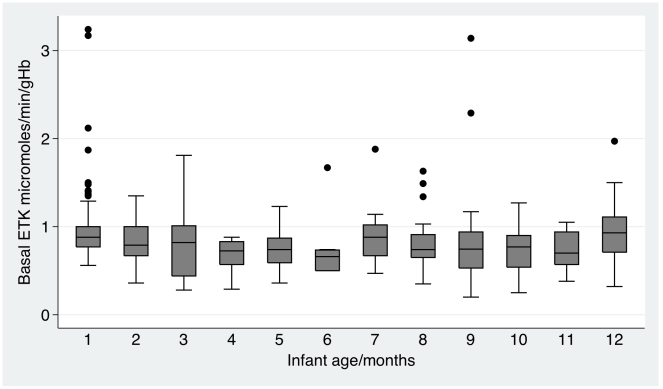
Boxplot of basal EKT per month of infant age. Median and 1^st^ and 3^rd^ quartiles and outliers.

**Figure 3:**
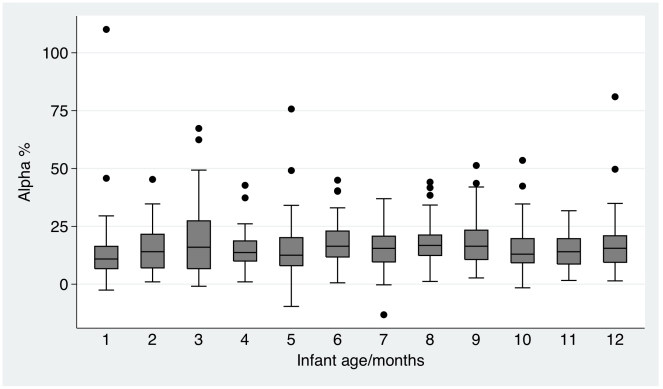
Boxplot of α per month of infant age. Median and 1^st^ and 3^rd^ quartiles and outliers.

Infant basal ETK<0.59 micromoles/min/gHb was associated with a significantly (P<0.02) higher frequency of infants presenting at ≥2 months of age, infant breastfeeding, death when ≥2 months old, a pneumonia discharge diagnosis, a lower frequency of prematurity discharge diagnosis, vomiting, dyspnoea, a lower pulse rate, fewer years of maternal schooling, a higher frequency of maternal Lao Sung ethnicity and maternal pork, chicken, fish or vegetables consumption since parturition (Table 1). α>31% was associated with a higher frequency of infants presenting at ≥2 months of age, of infant breastfeeding, vomiting, dyspnoea, cold fingers, maternal Lao Sung ethnicity, mothers being rice farmers and fewer years of maternal schooling. There was no apparent relationship between biochemical measurements of thiamin status and maternal food avoidance behaviour. There was a tendency for basal ETK to decline with infant age up to 6 months and then to increase (Fig. 2), whilst there was a tendency for α to rise in the first 3–6 months of age (Fig. 3). There was no obvious relationship between measures of biochemical thiamin deficiency and seasonality (data not shown).

Multivariate logistic regression of the above admission variables significant (P<0.02) on univariate analysis suggested that infant age ≥2 months (OR (95%CI) 11.49 (3.42–38.65), P<0.001) and fewer maternal years of schooling (OR (95%CI) (0.85 (0.78–0.93), P< = 0.001) were significantly independently associated with infant basal EKT<0.59 micromoles/min/gHb. For the α coefficient, multiple logistic regression of the above admission variables significant (P<0.02) on univariate analysis suggested that infant age ≥2 months (OR (95%CI) 4.21 (1.45–12.25), P = 0.008), the presence of cold fingers (OR (95%CI) 3.25 (1.61–6.53), P = 0.001), maternal occupation as rice farmer (OR (95%CI) 3.30 (1.33–8.21), P = 0.01) and fewer years of maternal schooling (OR (95%CI) 0.90 (0.82–0.99), P = 0.027) were significantly independently associated with α>31%.

**Figure 4:**
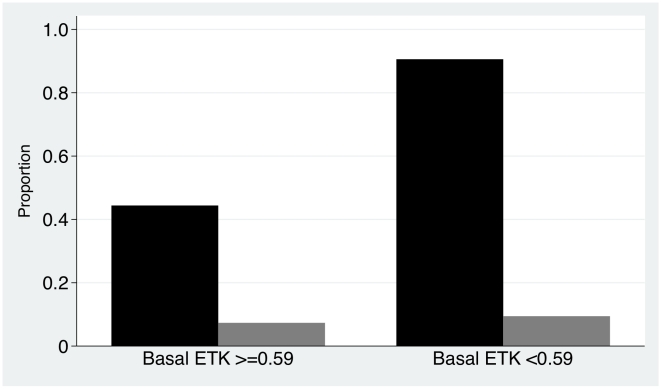
Proportion of infants aged ≥2 months and infant mortality. Categorized by basal ETK <0.59 and ≥0.59 in micromoles/min/gHB (n = 382 infants). Black bars – infants aged ≥2 months, grey bars – infants who died.

**Figure 5:**
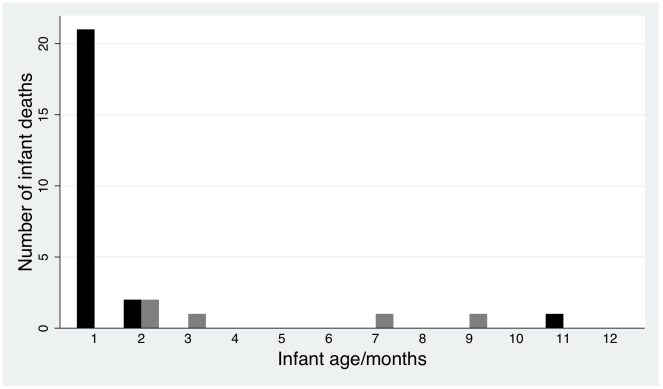
Number of infant deaths for each month age category. Black bars – death of infant with basal ETK ≥0.59 micromoles/min/gHB, grey bars - death of infant with basal ETK<0.59 micromoles/min/gHB.

Mortality was 5.5% but, among infants ≥2 months old, mortality was higher in those with basal ETK<0.59 micromoles/min/gHb (3/47, 6.4%) than in those with basal ETK≥0.59 micromoles/min/gHb (1/146, 0.7%, P = 0.045) (relative risk = 9.32 (95%CI 0.99 to 87.5)). (Figs. 4 & 5). Mortality was significantly (P<0.02) higher in younger infants, those who did not have a discharge diagnosis of diarrhoea or pneumonia, had a discharge diagnosis of prematurity, were not breast fed, did not receive rice water or chewed rice, had a faster pulse rate, lower body temperature, peripheral oedema, central cyanosis and respiratory distress. Overall, infants who died had lower α coefficient than those who lived. Basal ETK did not significantly differ between those who lived and died, but the sample size was considerably smaller (Table 2). There was no apparent relationship between mortality and the administration of thiamin after admission (P = 0.8). Multiple logistic regression (n = 746) of admission variables significantly associated (<0.02) with outcome on univariate analysis, suggested that breastfeeding (OR (95%CI) 0.29 (0.14–0.62) P = 0.001) and older age (OR (95%CI) 0.85 (0.74–0.97) P = 0.014) were associated with infant survival whilst respiratory distress (OR (95%CI) 5.04 (2.24–11.36) P<0.001) and higher pulse rate (OR (95%CI) 1.02 (1.00–1.04), P = 0.014) were independently associated with infant death.

**Table 2:** Mortality amongst 777 infants without clinical beriberi.

Variable	Survived	Died	P
Total	734	43	----
Infant age/months a	6 (0–12)	0 (0–12)	<0.0001
Infant age ≥2 months	496/734 (67.6)	8/43 (18.6)	<0.001
Infant male	424/727 (58.3)	22/43 (51.2)	0.36
Thiamin given after recruitment	143/726 (19.7)	7/39 (18.0)	0.79
Discharge diagnosis - Diarrhoea	236/734 (32.2)	2/43 (4.7)	<0.001
Discharge diagnosis - Pneumonia	187/734 (25.5)	4/43 (9.3)	0.017
Discharge diagnosis - Sepsis	86/734 (11.7)	8/43 (18.6)	0.18
Discharge diagnosis - Prematurity	70/734 (9.5)	12/43 (27.9)	<0.001
Breast fed	594/731 (81.3)	19/43 (44.2)	<0.001
Given rice water	322/731 (44.1)	5/43 (11.6)	<0.001
Given chewed rice	238/731 (32.6)	1/43 (2.3)	<0.001
Dyspnoea	289/731 (39.5)	15/43 (34.9)	0.54
Pulse/min b	120.8 (119.2–122.4) ^707^	142.7 (136.8–148.7)	<0.0001
Temperature °C b	37.6 (37.5–36.7) ^726^	36.7 (36.4–37.0)	<0.0001
Temperature >37.5 °C	398/726 (54.8)	8/43 (18.6)	<0.001
Respiratory rate/min b	49.9 (48.6–51.1) ^502^	53.9 (49.2–58.6) ^39^	0.09
Peripheral oedema	20/730 (2.7)	5/42 (11.9)	0.001
Central cyanosis	70/730 (9.6)	10/42 (23.8)	0.003
Peripheral cyanosis	139/730 (19.0)	12/42 (28.6)	0.13
Fingers cold	128/730 (17.5)	10/42 (23.8)	0.30
Respiratory distress	63/730 (8.6)	13/42 (31.0)	<0.001
% rice farmers	53/723 (7.3)	2/41 (4.9)	0.55
Lao Loum ethnicity	624/730 (85.5)	37/42 (88.1)	0.64
Years of schooling a	8 (0–16)	8 (0–16)	0.25
Food avoidance	303/732 (41.4)	18/43 (41.9)	0.95
Basal erythrocyte transketolase activity micromoles/min/gHB a	0.84 (0.2–3.14) ^352^	0.86 (0.36–3.24) ^29^	0.36
Basal transketolase activity <0.59 micromoles/min/gHB	46/352 (13.1)	5/29 (17.2)	0.53
Alpha % a	14.0 (−9.6–110.1)	8.6 (−13.2–45.3)	0.0002
Alpha>31%	39/734 (5.3)	1/43 (2.3)	0.39

a  =  median (range);

b  =  mean (95%CI). Dead = died in hospital or taken home severely ill, not expected to survive; Survived = discharged well or discharged against medical advice but expected to survive. Outcome unknown for one patient (not listed here).

## Discussion

This study suggests that a substantial minority of infants (13.4%) admitted without clinical evidence of beriberi had biochemical thiamin deficiency. Overall mortality was not associated with measures of biochemical thiamin deficiency, but the study was not powered to detect mortality differences. However, in support of the hypothesis that thiamin deficiency is an important cause of infant death in Vientiane, mortality was higher amongst those ≥2 months of age if they had low basal ETK (Fig. 4). A higher proportion of older infants, in comparison to those <2 months old, also had low basal ETK and α>31%. Largely forgotten work from the south Pacific and India [39], [40] suggested that a key clue to the importance of beriberi in a community was a secondary mortality peak at 3–4 months of age, after the high early mortality amongst neonates, as recently described from the Karen community displaced on the Thailand/Burma border [10].

As also noted for infantile beriberi, thiamin deficiency was associated with infant breastfeeding. The association between fewer years of maternal schooling and low basal ETK and α coefficient >31% suggests that aspects of maternal poverty and/or low levels of education in Vientiane may predispose their infants to thiamin deficiency. Aside from thiamin deficiency, infant mortality was associated with admission respiratory distress and non-breast feeding, suggesting that the care of these patients should be prioritized.

This study has important limitations. Two laboratories had to be used for ETK determinations and haemoglobin concentrations were not assayed in Oxford and therefore basal EKTA could not be expressed for these samples. The long sample storage period is unlikely to have affected α [35], [36] and if it did, it would have tended to underestimate thiamin deficiency [42]. It is likely that infants with thiamin deficiency have other clinically important nutritional deficiencies, such as vitamin A, riboflavin and folate. Lao children have a high frequency of stunting [18]. Seventy years ago a (unrandomised) clinical trial suggesting that thiamin supplementation in east London infants increased growth [27]. There is an urgent need to examine the inter-relationships between different nutrients, infection and Lao childhood growth and development.

Traditional prolonged post-partum Lao maternal food avoidances may lead to fatal wet beriberi in infants and neurological symptoms in nursing mothers [13], [18]. In the 2000 Lao National Health Survey 78% of women were recorded as observing food taboos after delivery and for a prolonged duration–a mean of 88 days [43]. Among 300 mother-infant pairs living on the outskirts of Vientiane, despite high levels of ante-natal care attendance (91%), 93% of women observed post-partum restricted diets and 96.6% of mothers were estimated to have inadequate thiamin intake [18]. One explanation for the persistence of infantile beriberi in Laos is that post-partum food avoidances last longer in Laos than elsewhere, such as in Malaysia and Tamil Nadu, where food avoidances generally cease ∼40 days after delivery [44], [45]. There was surprisingly no apparent relationship between either biochemical measurement of thiamin status and maternal food avoidance behavior [13], [18]. Indeed, the mothers of infants with low basal ETK had more frequently taken pork than those with higher basal ETK-surprisingly as pork is an important source of thiamin. However, the dietary assessment used here was crude and more detailed assessments are needed. The finding that Lao Sung maternal ethnicity was associated with a higher frequency of α>31% and basal ETK<0.59 micromoles/min/gHb suggests that more research is needed on ethnic and dietary risk factors for thiamin deficiency. Lao Sung ethnicity refers to mountain top people of diverse cultures and those near Vientiane may differ in dietary habits from those living in such more remote areas. That no infants with low basal ETK had a discharge diagnosis of prematurity reflects the relationship of basal ETK with age as all those with prematurity are, by definition, <2 months old.

Importantly, the clinical significance of biochemical thiamin deficiency in both those apparently healthy and in those ill without clinical beriberi remains unclear. Basal ETK is influenced by factors other than thiamin status-younger red cells have higher ETK and differences between patients could reflect variation in haematopoesis and red cell survival [46]. Presumably basal ETK would be higher in those with haemolysis associated with glucose-6-phosphatase deficiency. However, there was no significant difference in basal ETK between male and female infants. In addition there may, or may not, be different red cell transketolase isoenzymes in human erythrocytes, differing in their affinity for thiamin pyrophosphate [47].

Lao people have multiple risk factors for thiamin deficiency. The consumption of polished rice, alcohol and thiaminase-containing foods such as ‘paa dek’ (fermented fish paste), thiamin antagonists such as betel nut and the hard physical labour of rural rice farming are likely to be important in adults [5], [6]. Malaria and other fevers in pregnant and post-partum women may further predispose to beriberi in their infants by further depleting the short term body stores. It has also been suggested that biochemical thiamin deficiency predisposes to infection [48] and host genetic factors may be important in susceptibility as may occur in Wernicke's encephalopathy [49].

What do these results mean for Lao public health? There appears to be a significant but clinically unapparent burden of thiamin deficiency among sick infants admitted in Vientiane. Using the criteria of World Health Organization ([8] Table 7, α>25%), it would be described as a ‘mild’ public health problem. However, this definition may not be appropriate for infants [22] and given the ease and low cost of life-saving therapy it may be an important remedial public health issue. During the one year study 86 infants with suspected beriberi were admitted at Mahosot Hospital and between 2005 and 2008 ∼54 infants with beriberi were admitted/year with a mortality of 6%, despite thiamin therapy. At Luang Namtha provincial hospital in Northern Laos 3.4% of all children admitted 2008–2009 had a clinical diagnosis of beriberi (P. Douangdala, S. Inthalad, G. Slesak, unpublished data). There are anecdotal reports of beriberi from other areas of Laos (C. Perks, L. Srour, H. Barennes, T. Saito pers. comm.) but, given the wide ethnic and nutritional diversity in Laos, whether all communities are afflicted remains very unclear [12]. If highly milled glutinous rice in conjunction with post-partum food avoidance are key factors we might expect infantile beriberi to be, paradoxically, a more important public health problem in the relatively affluent Lao populations with access to rice mills, rather than the more remote poor communities who rely on hand pounding rice.

Aside from causing infant death, thiamin deficiency may have unappreciated longterm consequences on growth and neurological development. Among the infants on the Thailand/Burma border, McGready *et al.* [50] demonstrated delayed visual maturation type 1 and suggested thiamin deficiency as a possible cause. Follow up of Israeli infants who survived beriberi, due to substandard infant formula devoid of thiamin, suggests that thiamin deficiency was associated with subsequent delayed language development and epilepsy [51]. Whether epilepsy in some Lao patients is a consequence of infantile beriberi or clinically-cryptic thiamin deficiency requires investigation.

If sick infants are to be supplemented, these data suggest that breast fed infants aged ≥2 months, of mothers with few years of schooling should be the priority group. In sick infants thiamin should be administered parenterally to increase tissue thiamin levels rapidly. There is evidence that gastrointestinal absorption of thiamin is saturated at doses of >5 mg [5], suggesting that oral doses above this give limited, if any, benefit. How long oral supplementation of thiamin should be continued for is unclear. If thiamin is given to breastfed infants it should also be given to their mothers, but how and for how long is unknown. Similarly, whether dietary supplementation of pregnant and nursing Lao mothers with thiamin or thiamin-rich foods would improve child and maternal health in Laos remains unclear. Although 94.9% of Lao children were recorded as breastfed in the 2000 National Health Survey, only 23.6% of Lao infants aged under 4 months were recorded as exclusively breast fed [43]. It will be important not to allow concerns of thiamin deficiency to reduce the prevalence of breastfeeding. Although the cost of 100 mg parenteral thiamin plus syringe/needle in Vientiane is ∼1.5 $US, this is an appreciable amount in Laos where 74% of the population live on <2 $US/day [52]. Additional risks, such as injection abscesses and anaphylaxis, are present but rare [53] and there seems little evidence that thiamin supplementation increases the risk of cancer, although again there are very few data [54]. The high benefit and low cost of parenteral thiamin suggests that supplementation of older sick infants may be warranted. However, we would caution that the mortality difference presented here is based on only 4 deaths among 197 infants aged >2 months with basal ETK data with borderline relative risk and significance.

The most efficient method to determine whether the biochemical thiamin deficiency is clinically important and whether thiamin supplementation should be provided on admission for sick infants, or those with particular syndromes, would be a clinical trial. More data are needed on the importance of rice milling/pounding and soaking practices and food avoidance behaviour. The age distribution of infant mortality in Laos and their geographical and ethnic variations may give valuable clues as to the public health importance of beriberi and inform the need for preventative measures.

## Supporting Information

Checklist S1 STROBE checklist
(0.08 MB DOC)
